# Toward Patient Centricity: Why Do Patients With Inflammatory Bowel Disease Participate in Pharmaceutical Clinical Trials? A Mixed-Methods Exploration of Study Participants

**DOI:** 10.1093/crocol/otae019

**Published:** 2024-03-15

**Authors:** Virginia Solitano, Heather Prins, Meagan Archer, Leonardo Guizzetti, Vipul Jairath

**Affiliations:** Division of Gastroenterology, Department of Medicine, Western University, London, ON, Canada; Division of Gastroenterology, Department of Medicine, Western University, London, ON, Canada; Division of Gastroenterology, Department of Medicine, Western University, London, ON, Canada; Independent Researcher, London, ON, Canada; Division of Gastroenterology, Department of Medicine, Western University, London, ON, Canada; Department of Epidemiology and Biostatistics, Western University, London, ON, Canada

**Keywords:** ulcerative colitis disease, Crohn’s disease, clinical trials, experimental studies, design, trial framework, combined research methods, subject involvement

## Abstract

**Background:**

A better understanding of motivations to participate as well as recommendations to reduce barriers to enrollment may assist in design of future clinical trials.

**Methods:**

We developed a 32-item electronic questionnaire to explore motivations, experiences, and recommendations of inflammatory bowel disease patients, who had participated in pharmaceutical clinical trials in a tertiary center in Canada over the last decade. We employed a mixed-methods approach that integrates both quantitative and qualitative research methods.

**Results:**

We distributed a total of 69 e-mails with surveys and received 46 responses (66.6% response rate). Study participants were mostly male (27/46, 58.7%), non-Hispanic White (43/46, 93.5%), with a mean age of 45.5 years (SD 10.9). Most decided to participate in a clinical trial to benefit future patients (29/46, 63.0%). Half of the participants (23/46, 50.0%) reported they were worried about the possibility of receiving placebo, although the majority (29/46, 63.0%) understood they could improve on placebo. The most challenging aspect reported was the number and length of questionnaires (15/46, 32.6%), as well as the number of colonoscopies (14/46, 30.4%). Strategies recommended to increase enrollment were reduction of the chance of receiving placebo (20/46, 43.5%), facilitating inclusion of patients who have failed multiple therapies (20/46, 43.5%), allowing virtual visits (18/46, 39.1%), including subtypes of disease traditionally excluded from trials (16/46, 34.8%) and improving outreach to underrepresented populations (13/46, 28.3%). The vast majority (37/46, 80.4%) reported their experience of participation to be better than expected.

**Conclusions:**

These results should help inform the design of future clinical trials with a focus on patient-centricity.

## Graphical Abstract

**Graphical Abstract:**
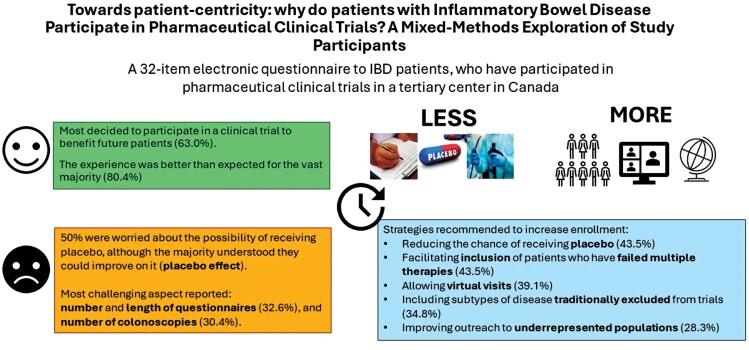

**Key Messages**

- Despite the increase in clinical development and availability of novel therapeutic options in inflammatory bowel disease (IBD), there has been a simultaneous decline in clinical trial enrollment rates.
- The FDA and other regulatory agencies have recognized the importance of patient engagement and the integration of patient perspectives in drug development.
- This survey provides novel insights into the demographics, motivations, knowledge, and experiences of patients with IBD, as well as recommendations to make trials more patient-centric.
- Key areas include reducing the likelihood of receiving placebos, minimizing the number of endoscopic procedures and questionnaires, expanding options of home-based clinical assessments, and including unrrepresented disease subtypes.

## Introduction

Between 1999 and 2019, the number of clinical trials initiated for Crohn’s disease (CD) and ulcerative colitis (UC) has more than doubled.^1^ While this increase in clinical development and availability of novel therapeutic options has transformed the care of patients living with inflammatory bowel disease (IBD), there has been a simultaneous decline in clinical trial enrollment rates.^1,2^ This decline is particularly concerning because the timely completion of clinical trial recruitment is the critical bottleneck to enable approval of new therapies, and it has been identified as a primary reason for discontinuing clinical trials prematurely.^3^

The reasons for declining enrollment rates are complex and multifactorial. These include the increasing availability of commercially available therapies, insufficiently trained investigators, as well as specifics of clinical trial design that include exposure to placebo, invasive procedures, and burden of participation.^4^ The average recruitment rate for CD clinical trials declined from 0.65 to 0.10 patients per site per month between 1998 and 2018, and for UC clinical trials, it fell from 0.32 to 0.13 patients per site per month during a similar time period.^3^ Practically this means that on average each clinical trial site will recruit only 1 participant per year thus requiring several hundred trial sites to complete a typical phase 3 trial.

Previous studies have explored the perceptions of patients regarding their involvement in clinical trials.^5^ A survey from India exploring the challenges encountered by investigators from various specialties (eg, oncology, cardiology, diabetes, etc.) in subject recruitment and retention revealed that commonly faced obstacles in recruiting subjects included the complex nature of the study protocol, limited patient awareness regarding clinical trials, and sociocultural concerns associated with trial participation.^6^ A study carried out by the Tufts Center for Drug Development reported the proportion of “non-core” procedures within clinical trials rose from 18% to 31% between 2002 and 2012.^7^ This increase in non-core procedures may contribute to the perception that participating in a clinical trial is burdensome, potentially influencing patients’ willingness to enroll.^7^

Clinical trials for IBD present distinct challenges, including extended washout periods for current therapies, prolonged corticosteroid exposure, repeated invasive procedures such as colonoscopy, the requirement for numerous hospital visits and assessments and the potential for exposure to a placebo. Collectively these factors may reduce willingness to participate in clinical trials. The US Food and Drug Administration and other regulatory agencies have recognized the importance of patient engagement and have developed guidelines and initiatives to encourage and support the integration of patient perspectives in the drug development process.^8^ Given the paucity of published research in IBD regarding patient engagement in clinical trials, we conducted a qualitative and quantitative study to explore the motivations and experiences of patients with IBD who had taken part in a pharmaceutical clinical trial as well as seek their recommendations for designing more patient-centric clinical trials in the future.

## Methods

### Study Population

Adult (≥18 years) patients with either UC or CD, previously enrolled in a pharmaceutical placebo-controlled clinical trial for IBD at University Hospital (London, Ontario, Canada) from January 1, 2013 to January 1, 2023 were eligible to take part. We focused on the last 10 years since this is the period where invasive procedures such as colonoscopy have been routinely implemented. Participants in this study did not receive any form of compensation for their involvement and participation was entirely voluntary. Our qualitative approach prioritized inductive research principles, emphasizing data saturation over predetermined sample sizes to capture comprehensive insights.^9^ Although no specific sample size was set, our initial goal was to collect data from 50 patients for dataset diversity. Participant identification involved meticulous pre-screening of past study enrollment logs spanning a decade.

### Ethical Approval

Ethical approval was obtained from Western Research Ethics Board and individual patient consent was obtained in order to participate in the survey.

### Survey Development

In December 2022, the development of the survey was initiated by researchers (V.S., H.P., M.A., V.J.) at London Health Sciences Center. This process was guided by a literature review of publications that documented experiences, motivations, and challenges in clinical trials from various other domains. Subsequently, the survey underwent a pilot test to assess its comprehensibility, readability, and timing, and it was subsequently revised to include the recommended modifications. The final electronic survey instrument included 32 questions; 26 were multiple-choice. Multiple responses were allowed for 8 questions, and there was an option to include alternative responses as free text under “other.” The qualitative research approach with free-text responses in this survey involved collecting and analyzing non-numeric data to gain a deeper understanding of participants’ perspectives, experiences, and opinions.

Within the survey framework, participants engaged with 7 thematic modules. The first module delved into demographic information through 8 questions, providing a comprehensive understanding of participants’ backgrounds. The second module explored participants’ motivations for joining a clinical trial and gathered reflections on their overall experiences using 5 questions. The third module aimed to gauge the participants’ level of knowledge and awareness regarding clinical trial design before their involvement, utilizing 2 questions to capture their pretrial perspectives. The fourth module delved into participants’ perceptions concerning the possibility of receiving a placebo during the clinical trial, employing 6 questions to unravel their thoughts on this crucial aspect. The fifth module (4 questions) focused on participants’ experiences with the burden of investigations, questionnaires, and procedures involved in the clinical trial. The sixth module aimed to assess participants’ understanding of the potential benefits and risks associated with clinical trial participation, utilizing 5 questions to capture nuanced insights. Finally, the seventh module centered on participants’ recommendations to make clinical trials more patient-centric. Through 2 questions, participants provided valuable perspectives on how to enhance the patient-centricity of future clinical trial designs based on their lived experiences (Table 1).

**Table 1.** Survey questions overview—Exploring motivations for participation in clinical trials: A questionnaire-based study of patients with inflammatory bowel disease. To note, survey encompasses both qualitative and quantitative elements to capture a comprehensive understanding from participants.

**Baseline demographic characteristics** *Gender* Male Female Other Prefer not to answer I identify as: __________________________________ *Age at the time of trial enrollment (years)* ___________ *Inflammatory Bowel Disease* Ulcerative Colitis Crohn’s Disease Prefer not to answer *Race* White First Nations, Inuit, or Metis Black or African American Asian or Pacific Islander Hispanic or Latino A race not listed here Prefer not to answer Please describe: __________________________________ *Highest education at the time of clinical trial enrollment* Less than a high school diploma or equivalent High school diploma or equivalent Registered apprenticeship or other trades certificate or diploma College, CEGEP or other non-university certificate or diploma University certificate or diploma below bachelor’s level Bachelor’s degree Post- graduate degree above bachelor’s level Prefer not to answer *Employment at the time of clinical trial enrollment* Full-time Part-time Not working due to my health issues Not working for “other” reasons Student Retired Stay-at-home-parent Prefer not to answer *Total yearly household income, before taxes, at the time of clinical trial enrollment* Less than $20 000 $20 000 to $49 999 $50 000 to $74 999 $75 000 to $99 999 $100 000 or more Prefer not to answer	**Reason for accepting clinical trial and overall experience** *Why did you participate in a clinical trial? (Please select all that apply)* I want to help future patients with IBD I was encouraged by my physician’s opinion and thoughts I had no other treatment options I thought I would receive better care I wanted to understand more about research Other Prefer not to answer Please specify: __________________________________ *Did you complete the clinical trial?* Yes No Prefer not to answer Did your condition improve? Yes No Unsure Prefer not to answer *Did you withdraw from the clinical trial?* Yes No Prefer not to answer *If yes, why did you withdraw your consent after entering the clinical trial? (Please select all that apply)* My disease did not respond to the treatment I had a side effect to treatment which resulted in withdrawal The burden of participation was too high The clinical trial was stopped Other Prefer not to answer Please specify: ____________________	**Level of knowledge and awareness of clinical trial design before participating in the clinical trial** *Overall, how would you rate your knowledge about clinical trials on a scale of 0 to 100, with 0 being no knowledge and 100 being extremely knowledgeable?* Please leave this question blank if you prefer not to answer. *Who provided the information regarding the clinical trial design to you? (Please select all that apply)* IBD physician IBD nurse/research coordinator Informed consent and written material Prefer not to answer **Perceptions on the possibility of receiving placebo in the clinical trial** *Did you understand that you might receive placebo? Please rank your level of understanding on a scale of 0 to 100, with 0 being no understanding and 100 being extremely understanding.* Please leave this question blank if you prefer not to answer. *Overall, how would rate your knowledge on the possibility of being assigned to placebo on a scale of 0 to 100, with 0 being no knowledge of the possibility and 100 being extremely knowledgeable of the possibility?* Please leave this question blank if you prefer not to answer. *Were you worried about the possibility of receiving placebo?* Yes No Unsure Prefer not to answer *Please rank your level of concern about the possibility of receiving placebo on a scale from 0 to 100, with 0 being no concern and 100 being extremely concerned.* Please leave this question blank if you prefer not to answer. *Did you understand that you could improve on placebo?* Yes No Unsure Prefer not to answer *Did you know about the existence of the placebo effect?* Yes No Unsure Prefer not to answer
**Experiences with the burden of investigations, questionnaires and procedures involved in the clinical trial** *Overall, how would you define your experience in the clinical trial:* Better than I expected Neutral Worse than I expected Unsure Prefer not to answer *If your experience was better than expected, which sentence best describes your feelings? (Please select all that apply)* I felt that I was receiving better care overall I learnt a lot about my condition I learnt a lot about research I built up a trust with my IBD team Not applicable Prefer not to answer *If your experience was worse than expected, which sentence best describe your feelings? (Please select all that apply)* My medical condition did not improve There were too many visits There were too many procedures I felt treated like a ‘guinea pig’ Not applicable Prefer not to answer *Were there aspects of the clinical trial which you found challenging? (Please select all that apply)* The informed consent forms were too complicated The length of steroid exposure The duration of washout period from the drug that I was on before entering the clinical trial The number of colonoscopies The number of blood tests The number of visits The frequency of study visits The number of questionnaires The length of questionnaires The length of time required for study visits Other Prefer not to answer Please specify: __________________________________	**Understanding of the potential benefits and risks of taking part in clinical trials** *Were you worried about taking part in the clinical trial?* Yes No Unsure Prefer not to answer *What was your main concern during the clinical trial?* The treatment not working The possibility of receiving placebo Side effects of the new treatment I had no concerns Other Prefer not to answer Please specify: __________________________________ *Would you recommend other patients with IBD to take part in the clinical trial?* Yes No Unsure Prefer not to answer *Do you believe that doctors and nurses treat patients taking part in clinical trials better than non-clinical trial patients?* Yes No Unsure Prefer not to answer *Would you consider taking part in a clinical trial again in the future?* Yes No Unsure Prefer not to answer	*Recommendations to make the clinical trial more patient-centric* *What would you change if you could participate in the design of a clinical trial? (Please select all that apply)* I would reduce the number of required procedures (ie, colonoscopies) I would replace colonoscopy will less invasive tests such as a CT or MRI scan I would reduce the number of required examinations (ie, blood tests, visits) I would include an option to have clinical trial visits at home to perform assessments, such as blood tests, to avoid having to travel to the hospital I would make the informed consent form easier to understand I would remove the placebo arm I would reduce the chance of receiving placebo I would reduce the length of the washout period I would not change anything Other Prefer not to answer Please specify: __________________________________ *What recommendation(s) would you make to improve the participation of patients with IBD in clinical trials? (Please select all that apply)* Improve education about clinical trials Improve outreach to underrepresented populations in clinical trials Allow inclusion of patient who have failed multiple therapies Facilitate inclusion of patients that have traditionally been excluded in clinical trials (eg, patients with ostomies, fistula. etc.) Reduce the chance of receiving placebo Reduce clinical trial complexity (frequency of visits, required procedures and examinations) Allow home administration of treatment under supervision Allow virtual visits to reduce travel to the clinical trial site Other Prefer not to answer Please specify: __________________________________

The survey took approximately 10–15 minutes to complete (based on pilot testing). Using the Total Design Method as offered by D.A. Dillman and aiming for a 60% return rate, potential participants were followed up 3–5 days and 2 weeks after the initial mail-out with a follow-up phone call.^10^ The purpose of the follow-up phone call was to ensure that potential participants had received the invitation and to clarify any outstanding questions from participants. Interested participants were presented the options to complete the questionnaire (online or over the phone), if they had not already completed the questionnaire using the link provided in the invitation. Sampling concluded upon achieving data saturation, marked by no new themes, considering factors like decreased responses, analysis time constraints, and staff availability.^9^

### Data Collection

We distributed surveys between July 17, 2023, and August 8, 2023, through online Research Electronic Data Capture software (RedCAP).

### Data Analysis

Electronic data were captured through a custom REDCap questionnaire. The same REDCap database stored the electronic consent (e-consent) forms completed by the participants and the IP address of the computer that the participant completed the e-consent form on, within its file repository. REDCap was administered through the Lawson Health Research Institute at the London Health Sciences Center.

Survey results were summarized descriptively, using counts and percentages for categorical responses and the mean, standard deviation, median, and interquartile range for continuous outcomes.

## Results

### Survey Response

We distributed a total of 69 e-mails with surveys and received 46 responses, for a 66.6% response rate.

### Demographic Characteristics of Participants

Of the 46 survey participants, 58.7% (27/46) were male (Table 2). Participants self-identified their race and ethnicity as follows: White (43/46, 93.5%), First Nations, Inuit, or Metis (0%), black or African American (1/46, 2.2%), Asian or pacific islander (0%), Hispanic or Latino (1/46, 2.2%) or reporting multiple racial or ethnic groups (1/46, 2.2%). The mean age was 45.5 years (SD 10.9). Both UC and CD were equally represented with 56.5% of individuals with UC. Regarding education, more than half of participants had a college, Collège d’enseignement général et professionnel (CEGEP) or other non-university certificate (17/46, 37.0%) or a high school diploma or equivalent (8/46, 17.4%). Seven participants (15.2%) had a Bachelor’s degree, 13.0% (6/46) had a university certificate or diploma below bachelor’s level and 8.7% had post-graduate degree above bachelor’s level (4/46). The majority of participants (33/46, 71.7%) had full-time employment and 2 individuals were working part-time. Only 3 participants reported that they were not working due to health issues. Among respondents, there were also 2 students, 2 retired individuals, and 3 stay-at-home parents.

**Table 2.** Demographics of survey respondents.

Characteristic	*n*	Statistic
Respondents, *N*	46	
Gender		
Male	27	58.7%
Female	18	39.1%
No response	1	2.2%
Age (years)		
*N*		39
Mean (SD)		45.5 (10.9)
Median (Q1, Q3)		46 (39, 51)
IBD Type		
UC	26	56.5%
CD	20	43.5%
Racial/Ethnic group		
White	43	93.5%
Black or African American	1	2.2%
Hispanic or Latino	1	2.2%
Multiple	1	2.2%
Educational achievement		
Less than a high school diploma or equivalent	2	4.3%
High school diploma or equivalent	8	17.4%
Registered apprenticeship or other trades certificate or diploma	2	4.3%
College, CEGEP or other non-university certificate or diploma	17	37.0%
University certificate or diploma below bachelor's degree	6	13.0%
Bachelor degree	7	15.2%
Post-graduate degree above bachelor's degree	4	8.7%
Employment category		
Full-time	33	71.7%
Part-time	2	4.3%
Not working due to own health issues	3	6.5%
Student	2	4.3%
Retired	2	4.3%
Stay-at-home parent	3	6.5%
Prefer not to answer	1	2.2%
Income category		
< $20 000	1	2.2%
$20 000 to $49 999	5	10.9%
$50 000 to $74 999	14	30.4%
$75 000 to $99 999	7	15.2%
≥$100 000	11	23.9%
Prefer not to answer	7	15.2%
No answer	1	2.2%

Approximately 40% of respondents reported an income of more than $75 000/year (7/46 with $75 000 to $99 999/year, and 11/46 with equal or more than $100 000/year), with only 1 patient reporting a salary of less than $20 000 per year. Eight participants preferred not to declare their income (Supplementary Figure S1).

### Reason for Accepting Clinical Trial and Overall Experience

Most participants (29/46, 63.0%) decided to participate in a clinical trial to help future patients with IBD and 54.3% (25/46) were encouraged by their physician’s opinion and thoughts. Almost half (20/46, 43.5%) of respondents reported that they had no other treatment options available other than taking part in a clinical trial. Approximately 40% perceived that they would receive better care (19/46, 41.3%). On free text comments, some participants reported that they took part due to lack of drug coverage (Supplementary Figure S2).

More than 75% (35/46) of participants completed the trial they were enrolled in, and the majority reported (32/46, 69.6%) that their condition improved. Approximately 20% (8/46, 17.4%) of participants reported that they had withdrawn from the clinical trial. The main reason was lack of response to the investigational agent and worsening disease over the trial (8/8), with one subject withdrawing due to family planning.

### Level of Knowledge and Awareness of Clinical Trial Design Before Participating in the Clinical Trial

The average patient rating about their knowledge of clinical trials before deciding to participate on a scale from 0 (no knowledge) to 100 (very knowledgeable) was 64.6 (SD 29.7). Participants reported that information was delivered mainly by the IBD nurse/research coordinator (89.13%), and their IBD physician (56.52%). Informed consent and written material were perceived to be informative for only half of the participants (Table 3).

**Table 3.** Level of knowledge and awareness of clinical trial design before participating in the clinical trial.

Question	*n*	Statistic
Respondents, *N*	46	
Overall, how would you rate your knowledge about clinical trials?		
*n*		45
Mean (SD)		64.6 (29.7)
Median (Q1, Q3)		75 (50, 86)
Who provided the information regarding the clinical trial design to you? (Please select all that apply)		
IBD physician	26	56.5%
IBD nurse/research coordinator	41	89.1%
Informed consent and written material	25	54.3%
Prefer not to answer	0	0.0%

### Perceptions on the Possibility of Receiving Placebo in the Clinical Trial

The average patient rating about their understanding of the possibility of receiving placebo on a scale from 0 (no understanding) to 100 (extremely understanding) was extremely high, 97.6 (SD 8.2). Accordingly, participants rated their knowledge on the possibility of being assigned to placebo on a scale from 0 (no knowledge) to 100 (extremely knowledgeable) 95.64 (SD 11.7). Half of the participants (23/46, 50.0%) reported that they were worried about the possibility of receiving placebo and overall, the level of concern on a scale from 0 (no concern) to 100 (extremely concerned) was medium (54.2, SD 37.2). Importantly, 63.0% of participants understood they could improve on placebo (placebo effect).

### Experiences With the Burden of Investigations, Questionnaires, and Procedures Involved in the Clinical Trial

The overall experience of taking part in the clinical trial was positive for the majority of participants (37/46, 80.4%). Only one participant said the experience turned out to be worse than expected. For those who stated their experience was better than expected, the reasons behind were improved trust with the IBD team (33/46, 71.7%), better overall care (31/46, 67.4%), improved knowledge about their disease (28/46, 60.9%) and about research in general (20/46, 43.5%).

For the single participant who indicated their experience as worse than expected indicated this was due to lack of improvement in the medical condition.

The most critical aspects of the clinical trial participants found challenging were the number and length of questionnaires (32.6% and 28.3%, respectively) and the number of colonoscopies required (14/46, 30.4%). Less than 20% (7/46) indicated blood tests and study visits as troublesome, whereas the length of time required for study visits was reported as problematic for only 3 (6.5%) participants. Seven respondents (15.2%) indicated the duration of washout period from the drug before entering the clinical trial was challenging, whereas only 4 (8.7%) participants found the length of steroid exposure challenging. When asked to explain their thoughts using the free text, most participants pointed out that distance from clinical trial center became cumbersome over time (Table 4).

**Table 4.** Experiences with the burden of investigations, questionnaires and procedures involved in the clinical trial.

Question	Frequency	Percent
Respondents, *N*	46	
Overall, how would you define your experience in the clinical trial?		
Better than I expected	37	80.4%
Neutral	8	17.4%
Worse than I expected	1	2.2%
If your experience was better than expected, which sentence best describes your feelings? (Please select all that apply)		
I felt that I was receiving better care overall	31	67.4%
I learnt a lot about my condition	28	60.9%
I learnt a lot about research	20	43.5%
I built up a trust with my IBD team	33	71.7%
Not applicable	0	0%
Prefer not to answer	0	0%
If your experience was worse than expected, which sentence best describe your feelings? (Please select all that apply)		
My medical condition did not improve	1	2.2%
There were too many visits	0	0%
There were too many procedures	0	0%
I felt treated like a “guinea pig”	0	0%
Not applicable	0	0%
Prefer not to answer	0	0%
Were there aspects of the clinical trial which you found challenging? (Please select all that apply)		
The ICF was too complicated	0	0.0%
The length of steroid exposure	4	8.7%
The number of colonoscopies	14	30.4%
The duration of washout period	7	15.2%
The number of blood tests	7	15.2%
The number of visits	5	10.9%
The frequency of visits	5	10.9%
The number of questionnaires	15	32.6%
The length of questionnaires	13	28.3%
The length of time required for study visits	3	6.5%
Other	6	13.0%
Prefer not to answer	2	4.3%

### Understanding of the Potential Benefits and Risks of Taking Part in Clinical Trials

Overall, only 17.4% (8/46) of participants reported that they were worried about taking part in the clinical trial. The main reason for concern during the clinical trial was treatment lack of efficacy (28/46, 60.9%), rather than the possibility of experiencing side effects (11/46, 23.9%) and the possibility of receiving placebo (5/46, 10.9%).

Almost all participants would recommend other patients with IBD to take part in the clinical trial (45/46, 97.8%), and 91.3% (42/46) would consider taking part in a clinical trial again in the future if the opportunity arose. Approximately one-third of respondents (14/46, 30.4%) believed that doctors and nurses treat patients taking part in clinical trials better than nonclinical trial patients.

### Recommendations to Make the Clinical Trial More Patient-Centric

**Figure 1.**
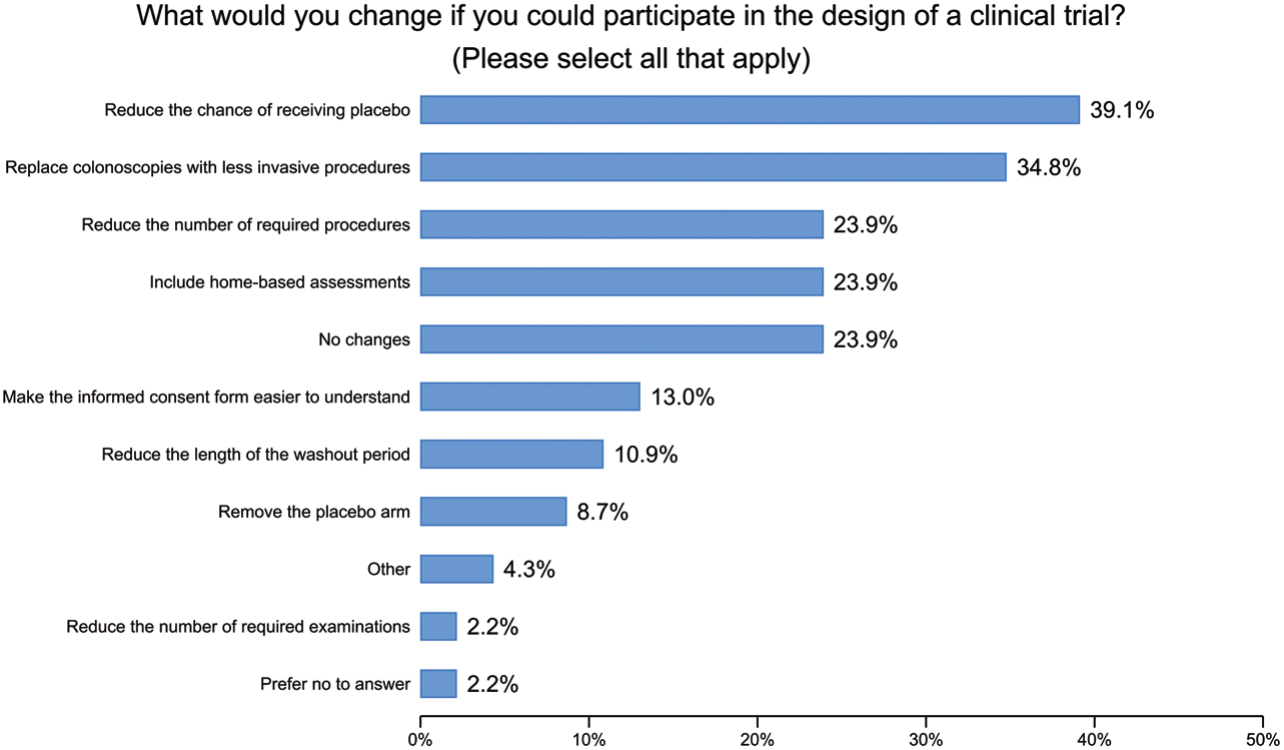
Question: What would you change if you could participate in the design of a clinical trial? *(Please select all that apply).*

When asked “What would you change if you could participate in the design of a clinical trial?,” reducing the chance of receiving placebo was the primary recommendation by almost 40% of participants and 9% suggested removing the placebo arm altogether (Figure 1). Most responders also proposed reducing the number of required procedures, with 34.8% (16/46) proposing to replace colonoscopies with less invasive procedures. Including an option to have clinical trial visits at home to perform remote assessments without having to travel to the hospital was suggested by 23.9% (11/46) of participants.

Towards a more patient-centric approach, we asked participants which recommendations they would make to improve the participation of patients with IBD in future clinical trials (Supplementary Figure S3). The single most important issue to participants was to reduce the chance of receiving placebo (47%, *n* = 15 with a single response to question). For those choosing 2 responses (*n* = 8), the most important issues were to allow virtual visits to reduce travel (25%), reduce the chance of receiving placebo (19%), and allow the inclusion of patients who have failed multiple therapies (19%). When 5 items were selected, making the trial more inclusive to patients was among the highest priority (20%).

**Figure 2.**
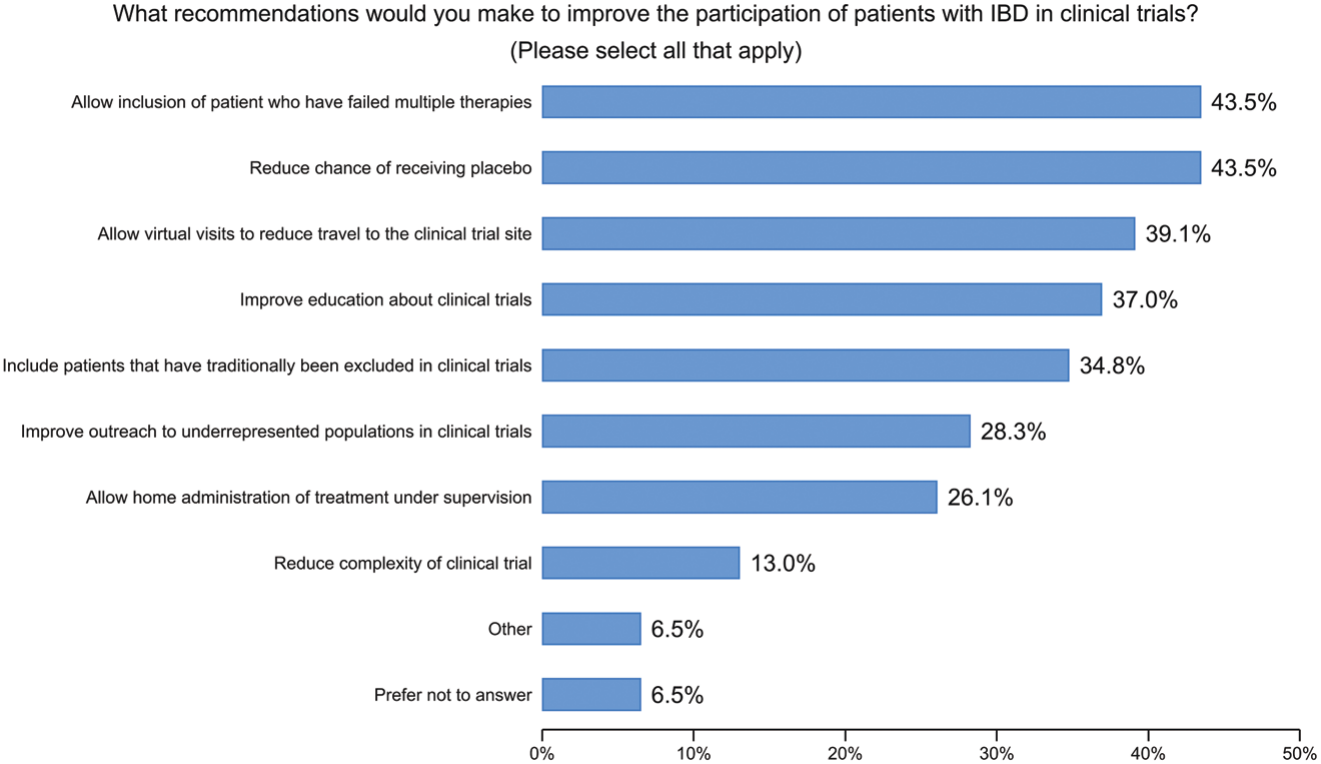
Question: What recommendation(s) would you make to improve the participation of patients with inflammatory bowel disease in clinical trials? *(Please select all that apply).*

In addition, they would facilitate the inclusion of patients who have traditionally been excluded in clinical trials (eg, patients with ostomies, fistula, etc.; 16/46, 34.8%) and improve the outreach to underrepresented populations in clinical trials (13/46, 28.3%). A substantial percentage of participants (18/46, 39.1%) overall would address logistical constraints such as travel time by allowing home administration of treatment under supervision and/or virtual visits (Figure 2). Table 5 summarizes all recommendations to make the clinical trial more patient-centric.

**Table 5.** Recommendations to make the clinical trial more patient-centric.

Question	Frequency	Percent
Respondents, *N*	46	
What would you change if you could participate in the design of a clinical trial? (Please select all that apply)		
Reduce the number of required procedures	11	23.9%
Replace colonoscopies with less invasive procedures	16	34.8%
Reduce the number of required examinations	1	2.2%
Include home-based assessments	11	23.9%
Make the informed consent form easier to understand	6	13.0%
Remove the placebo arm	4	8.7%
Reduce the chance of receiving placebo	18	39.1%
Reduce the length of the washout period	5	10.9%
No changes	11	23.9%
Other	2	4.3%
Prefer no to answer	1	2.2%
What recommendation(s) would you make to improve the participation of patients with IBD in clinical trials? (Please select all that apply)		
Improve education about clinical trials	17	37.0%
Improve outreach to underrepresented populations in clinical trials	13	28.3%
Allow inclusion of patient who have failed multiple therapies	20	43.5%
Include patients that have traditionally been excluded in clinical trials	16	34.8%
Reduce chance of receiving placebo	20	43.5%
Reduce complexity of clinical trial	6	13.0%
Allow home administration of treatment under supervision	12	26.1%
Allow virtual visits to reduce travel to the clinical trial site	18	39.1%
Other	3	6.5%
Prefer not to answer	3	6.5%

## Discussion

Patient engagement has gained increasing recognition and importance in the development and execution of clinical trials.^11,12^ Not only do patients benefit but higher engagement also contributes to the overall success of the trial and the development of new therapies.^13^ To the best of our knowledge this is the first survey conducted specifically in patients with IBD who have actually participated in pharmaceutical trials to document their experiences and recommendations for the design of future clinical trials.

To note, awareness of the possibility of receiving placebo was evident with about half explicitly expressing concern about this prospect, but also indicating understanding of the placebo effect among respondents. Despite the availability of several advanced therapeutics, placebo-controlled trials are still considered the gold standard for IBD trials. The importance of placebo for measuring safety in pivotal trials is acknowledged.^14^ However, ethical concerns and potential harm associated with prolonged placebo exposure are valid.^15^ Several strategies have been employed to reduce the chance and length of exposure to placebo including asymmetrical randomization, inclusion of an active comparator arm, and early transition to open-label therapy after induction in placebo nonresponders. Omitting exposure to placebo would increase patients’ readiness to participate, and may enhance recruitment rates, even though differences in screening failure rates to conventional placebo-controlled trials need to be explored. Alternative clinical trial methods such as Bayesian designs can reduce sample size requirements and placebo exposure.^16^ This methodology is underutilized in IBD and the large amount of prior data available on placebo rates makes this design more feasible.^17,18^ In a proof-of-concept study in CD, Hueber et al. used a Bayesian design augmented with historical information obtained from a meta-analysis to reduce the number of patients assigned to the placebo group.^19^ To overcome the disadvantage of historical controls, the information was appropriately down-weighted according to between-trial variability. At present, this low adoption of Bayesian methods is likely due to a lack of familiarity with Bayesian methods,^20^ as well as a lack of willingness to take deviate from frequentist approaches to drug development due to perceived risk. Other methods include the use of external control arms as an alternative to placebo.^21,22^ However, establishing historical control groups is challenging in this field due to the frequent introduction of new treatments, and increasing inclusion of patients who have failed multiple therapies into clinical trials.^23^

Over the last decade, endoscopy with central assessment (with or without biopsies from histology) has become a gold standard for regulatory approval of novel therapies.^24^ Endoscopic evaluation is integral to trial eligibility as well as primary endpoint assessment as part of the composite Mayo Clinic score for UC and co-primary endpoint for the Simple Endoscopic Score for Crohn’s Disease (SES-CD).^25,26^ This has led to a reduction in placebo rates and ensured patients with active disease are enrolled in clinical trials. In a typical 52-week trial, endoscopic evaluation is needed at baseline, end of induction, and end of maintenance.^27^ Reducing the burden of endoscopy was raised as a key area to improve further trials in this study. Strategies could include allowing routine clinical care videos captured within recent weeks for baseline assessment, provided they are of sufficient quality and cinematography for centralized scoring, as well as adjunctive screening tools such as cross-sectional imaging or intestinal ultrasound.

Balancing the pursuit of comprehensive data with the well-being of participants is key to refining clinical trial protocols and ensuring a more patient-centered approach. Reducing the high burden of questionnaires was also a key recommendation from patients in this study. While there is temptation to include multiple patient-reported outcomes (PROs) such as fatigue, urgency, sleep cycles, and work productivity in order to capture a broad patient experience, it is important to critically review this burden as trial programs are designed.^28^ This is relatively easy to implement since many PROs are not required for regulatory purposes and effectively become marketing tools as secondary or exploratory endpoints.^29,30^ Critical review of the number of PROs included within a trial program is essential and this burden should also be assessed by ethics committees. Furthermore, adopting user-friendly digital platforms and intuitive interfaces can enhance the ease with which patients provide essential information about their experiences.^31^

The observation that 39% of participants could overcome logistical challenges, such as travel time, through home administration of treatment under supervision and virtual visits underscores the importance of decentralized trials. Embracing decentralized approaches in clinical trials can revolutionize the landscape by increasing accessibility and participation rates. Home administration not only eases the burden on participants but could also serve to enhance the diversity of the participant pool, ensuring a more representative sample.^32^ Virtual visits contribute to the flexibility and convenience that many individuals seek, ultimately fostering greater engagement and compliance with the trial protocol.^33^ By reducing the need for frequent in-person visits, decentralized trials not only address logistical constraints but also promote a patient-centric model that aligns with the evolving expectations and preferences of participants, ultimately advancing the efficiency and inclusivity of clinical research.

The baseline demographics highlighted that over 40% of participants reported an income surpassing $75 000/year. Financial stability can undoubtedly facilitate participation by providing resources to cover incidental costs associated with the trial and time away from employment.^34^ However, this observation highlights how clinical trials may not reach underrepresented populations from a financial perspective or indeed in terms of race/ethnicity, echoing the conclusions drawn from prior studies.^35^

This study highlights the key role of altruism in motivating individuals to join clinical trials, driven by their recognition of medical research’s impact on advancing IBD treatments. Healthcare providers play a crucial role in guiding patients’ decisions on trial participation, emphasizing the importance of informed providers for an enhanced healthcare experience.^36^ Most subjects (63%) admitted participating in a clinical trial to benefit future patients with IBD. Taken together, this underscores that participants in clinical trials place a high degree of trust in both their care providers but also in the quality design of the trial from which they may never benefit.^37^ Along these lines, a significant percentage of patients exhibit limited knowledge about clinical trials, with IBD nurses and research coordinators serving as their primary source of information.

Expanding eligibility into clinical trials is an important focus for researchers. Most clinical trials are excluding patients with subtypes of disease such as active peri-anal fistula, ostomies, and stricturing disease.^38^ Collectively this excludes a substantial proportion of participants who also may serve to benefit from future therapies. Development of rigorous endpoints for clinical trials in these subpopulations is key to enhance trial eligibility. Furthermore, limits on the number of prior failed therapies also pose a challenge to patients who have exhausted all available commercial therapies. Additionally, respondents highlighted both areas as key strategies to make clinical trials more inclusive. The survey participants suggest multiple avenues to improve patient understanding, including educational efforts by the centers in charge, site investigators, and patient organizations. The involvement of patients in study design is an intriguing proposal, enabling their insights to shape trial parameters and minimize inconvenient study details and procedures.^8^

Our study, conducted within a globally renowned center widely acknowledged for running numerous clinical trials in IBD, represents a distinctive strength of this paper. This unique setting not only enhances the credibility of our findings but also positions our research at the forefront of advancements in IBD treatment. Moreover, our research is underpinned by a robust methodology, ensuring the collection of high-quality data, while the inclusion of qualitative research components enriches the depth of our analysis, providing a comprehensive understanding of participants’ experiences. However, we acknowledge certain limitations. The relatively modest sample size may impact the statistical power and generalizability of our findings. Patient diversity might be limited, affecting the broader applicability of results, and the geographical focus may limit external validity. Additionally, the method of study conduct, relying on email and phone, could potentially exclude certain patient groups, introducing a source of bias.

In conclusion, this study provides novel insights into the demographics, motivations, knowledge, and experiences of patients with IBD who have taken part in pharmaceutical clinical trials, as well as recommendations to improve their design to make them more patient-centric. Key areas included designing clinical trials to reduce the likelihood of receiving placebos, minimizing the number of endoscopic procedures and questionnaires, expanding the options of home-based clinical assessments, and including disease subtypes. These insights offer valuable guidance for researchers, healthcare providers, and policymakers in their efforts to make clinical trials more patient-centric and effective in advancing treatments for IBD.

## Supplementary Material

otae019_suppl_Supplementary_Figure_1

otae019_suppl_Supplementary_Figure_2

otae019_suppl_Supplementary_Figure_3

otae019_suppl_Supplementary_Material

## Data Availability

The data underlying this article will be shared on a reasonable request to the corresponding author.
