# Supplementary figures and images for: Toward Patient Centricity: Why Do Patients With Inflammatory Bowel Disease Participate in Pharmaceutical Clinical Trials? A Mixed-Methods Exploration of Study Participants
Source: Crohns Colitis 360. 2024 Mar 15;6(2):otae019. doi: 10.1093/crocol/otae019 (PMC11003535; doi:10.1093/crocol/otae019)

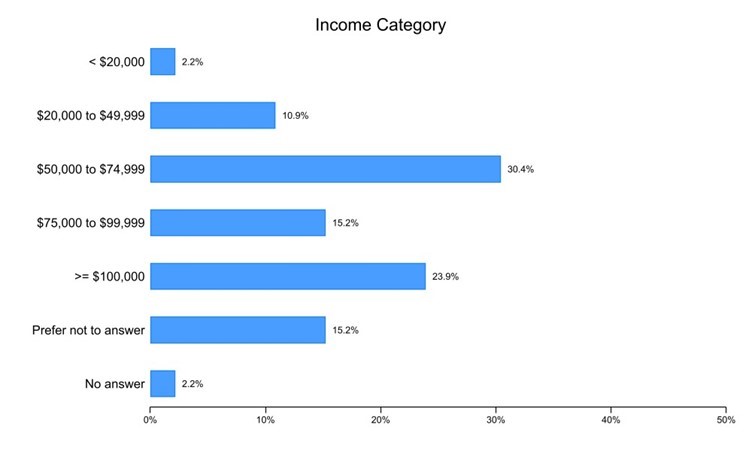

Supplement: otae019_suppl_Supplementary_Figure_1 [file otae019_suppl_supplementary_figure_1.jpeg]

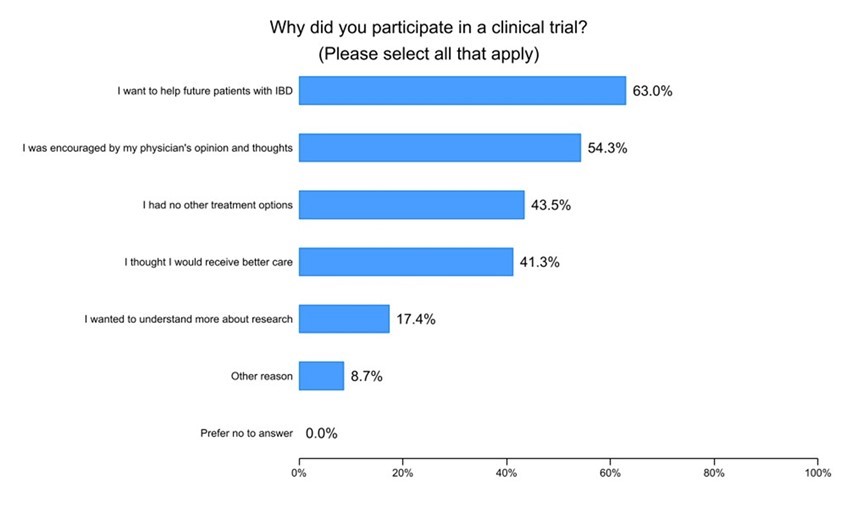

Supplement: otae019_suppl_Supplementary_Figure_2 [file otae019_suppl_supplementary_figure_2.jpeg]

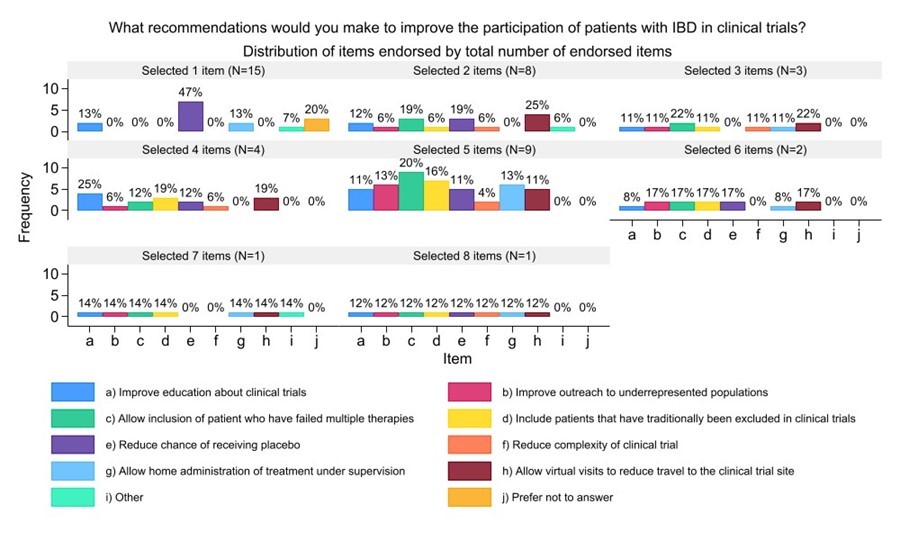

Supplement: otae019_suppl_Supplementary_Figure_3 [file otae019_suppl_supplementary_figure_3.jpeg]
