# Supplementary material for: Toward Patient Centricity: Why Do Patients With Inflammatory Bowel Disease Participate in Pharmaceutical Clinical Trials? A Mixed-Methods Exploration of Study Participants
Source: Crohns Colitis 360. 2024 Mar 15;6(2):otae019. doi: 10.1093/crocol/otae019 (PMC11003535; doi:10.1093/crocol/otae019)
Supplement: otae019_suppl_Supplementary_Material [file otae019_suppl_supplementary_material.docx]

**SUPPLEMENTARY MATERIAL**

Supplementary Figure 1. Income category

Supplementary Figure 2. Question: Why did you participate in a clinical trial? *(Please select all that apply)*

Supplementary Figure 3. Question: What recommendation(s) would you make to improve the participation of patients with IBD in clinical trials? (Please select all that apply). How many items are endorsed by a respondent (they selected just 1 item only, all the way up to 8 items in this case).

*Supplementary Figure 3 illustrates how many items are endorsed by a respondent (they were allowed to select just 1 item only, all the way up to 8 items). Within those categories, the bars show the proportion of each selected response.*
